# Microstructure, Tensile Properties, and Fracture Toughness of an In Situ Rolling Hybrid with Wire Arc Additive Manufacturing AerMet100 Steel

**DOI:** 10.3390/mi15040494

**Published:** 2024-04-03

**Authors:** Lei Lei, Linda Ke, Yibo Xiong, Siyu Liu, Lei Du, Mengfan Chen, Meili Xiao, Yanfei Fu, Fei Yao, Fan Yang, Kun Wang, Baohui Li

**Affiliations:** Shanghai Engineering Technology Research Center of Near-Net-Shape Forming for Metallic Materials, Shanghai Spaceflight Precision Machinery Institute, Shanghai 201600, China; leileix5@126.com (L.L.);

**Keywords:** in situ rolling, wire arc additive manufacturing, AerMet100 steel, tensile property, fracture toughness

## Abstract

As a type of ultra-high strength steel, AerMet100 steel is used in the aerospace and military industries. Due to the fact that AerMet100 steel is difficult to machine, people have been exploring the process of additive manufacturing to fabricate AerMet100 steel. In this study, AerMet100 steel was produced using an in situ rolling hybrid with wire arc additive manufacturing. Microstructure, tensile properties, and fracture toughness of as-deposited and heat-treated AerMet100 steel were evaluated in different directions. The results reveal that the manufacturing process leads to grain fragmentation and obvious microstructural refinement of the AerMet100 steel, and weakens the anisotropy of the mechanical properties. After heat treatment, the microstructure of the AerMet100 steel is mainly composed of lath martensite and reversed austenite. Alloy carbides are precipitated within the martensitic matrix, and a high density of dislocations is the primary strengthening mechanism. The existence of film-like austenite among the martensite matrix enhances the toughness of AerMet100 steel, which coordinates stress distribution and restrains crack propagation, resulting in an excellent balance between strength and toughness. The AerMet100 steel with in situ rolling is isotropy and achieves the following values: an average ultimate strength of 1747.7 ± 16.3 MPa, yield strength of 1615 ± 40.6 MPa, elongation of 8.3 ± 0.2% in deposition direction, and corresponding values in the building direction are 1821.3 ± 22.1 MPa, 1624 ± 84.5 MPa, and 7.6 ± 1.7%, and the K_IC_ value up to 70.6 MPa/m^0.5^.

## 1. Introduction

AerMet100 is a high alloyed secondary hardening steel with ultra-high strength, featuring remarkable plasticity and fatigue strength [1,2]. The exceptional properties of this steel make it an excellent choice for the manufacture of high-performance components in the aerospace and military industries [3,4,5]. In recent years, extensive research has been carried out on AerMet100 steel. The microstructure of AerMet100 steel consists of lath martensite, small amounts of residual/reversed austenite, and carbides precipitated in the martensite matrix [6,7]. The crystal morphology of as-deposited laser additive manufacturing AerMet100 steel is a columnar crystal with a size of 100–600 μm, and the mechanical properties are anisotropic [8]. After heat treatment, the prior austenite grains transformed into martensite packets, and the columnar crystals transformed into equiaxed crystals to improve the material properties [9]. The average size of AerMet100 grains fabricated using wire arc additive manufacturing (WAAM) with in situ rolling can be refined from about 150–600 µm to 20 μm after heat treatment [10]. In AerMet100 ultra-high-strength steel, the lath martensite is rich in dislocations due to high lattice strain caused by martensitic transformation after high-temperature quenching, and during the tempering process, carbides are precipitated, resulting in pinning dislocations, which is one of the most important factors in the strength of AerMet100 steel [11]. As a secondary precipitation aging steel, the tempering temperature and time will affect the type and size of precipitates, thus affecting the performance of the material [12]. M_2_C carbides can be formed by the enrichment of supersaturated carbon atoms and the substitution of Mo and Cr atoms for Fe atoms during the tempering process [13]. AerMet100 steel achieves the best combination of strength, plasticity, and toughness at a tempering temperature of 482 °C because the continuous film of reversed austenite starts to precipitate at the martensite boundaries, and the M_2_C carbide can grow to a suitable size [6,14]. On the basis of the above-mentioned study, the temperature of 482 °C was also chosen for the annealing treatment in this experiment in order to obtain favorable mechanical properties.

Due to the fact that AerMet100 steel presents challenges in terms of processing and forming [15], people have been exploring the process of additive manufacturing to fabricate AerMet100 steel, such as laser cladding [16], laser additive manufacturing (LAM) [17], laser direct energy deposition (LDED) [18] and friction stir processing (FSP) [18]. As a kind of hybrid deposition and microrolling (HDMR) method, in situ rolling with wire arc additive manufacturing is a novel additive manufacturing technique that combines microrolling with WAAM to produce large components. The TC4 [19] alloy produced using in situ rolling has a higher dislocation density and larger α-bars than the alloy produced using WAAM, which improves the tensile strength and plasticity of TC4. Al-Mg4.5Mn alloys [20] produced using in situ interlayer rolling resulted in significant grain refinement; the mean and maximum grain dimensions were reduced from 59 to 23 μm and 172 to 98 μm, respectively, and the number and size of defects were significantly reduced than those produced using WAAM. Zhao [21] found that applying external force to the AerMet100 steel surface leads to a notable refinement of the martensitic slats and the generation of significant compressive residual stresses. Lu [10] investigated the grain refinement mechanism and the microstructure evolution of in situ rolling hybrid WAAM fabricated AerMet100 steel, which obtained a fully refined and segregation-free zone. Zhi [22] et al. introduced deep residual compressive stresses in the surface layer of AerMet100 steel, which refines the surface structure and reduces fatigue notch sensitivity, thus significantly improving fatigue life and fatigue crack initiation/expansion resistance.

Fracture toughness is a critical material property that demonstrates the structural integrity and reliability of the material, taking into account the overall effect of applied loads in the event of failure [23,24]. The standard fracture test procedure demands preparation of the specimen, pre-fatigue of the notch, measurement of the fatigue crack growth rate, and evaluation of the original data [25]. Some studies on additive manufacturing have shown that material fracture toughness is anisotropic in the horizontal and vertical directions [26,27]. Tempering treatment improved the fracture toughness but not the anisotropy [28]. Lu [10] investigated the fracture toughness of heat-treated AerMet100 steel fabricated using WAAM with in situ microrolling and demonstrated that the fracture toughness under plane strain conditions is connected to the homogenization of the composition, which enhanced the K_IC_. Ran [8] investigated the fatigue crack growth (FCG) characteristics in the deposition and building directions of AerMet100 steel produced using laser additive manufacturing and revealed that the FCG of as-deposited AerMet100 steel is anisotropic with a lower crack resistance along the direction of deposition. The fatigue crack growth resistance of the heat-treated specimens has been improved. Meanwhile, the fatigue crack growth rate of steel can be effectively reduced by introducing thicker film-like reversed austenite. Jyoti Suryawanshi [29] et al. weakened the anisotropy of the fracture toughness of martensitic aging steels by 90° rotation between successive layers during selective laser melting (SLM) printing. Up to now, there has been a lack of comparative studies on the fracture toughness of AerMet100 steel in the deposition direction and building direction.

Therefore, the main objective of this study is to understand better the properties of AerMet100 steel processed using wire arc additive manufacturing hybrid with in situ rolling. In particular, the microstructure and tensile properties of the as-deposited and heat-treated AerMet100 steel were investigated, and the plane strain fracture toughness of the AerMet100 steel was investigated considering the different directions.

## 2. Materials and Methods

### 2.1. Material Manufacturing and Heat Treatment

**Figure 1:**
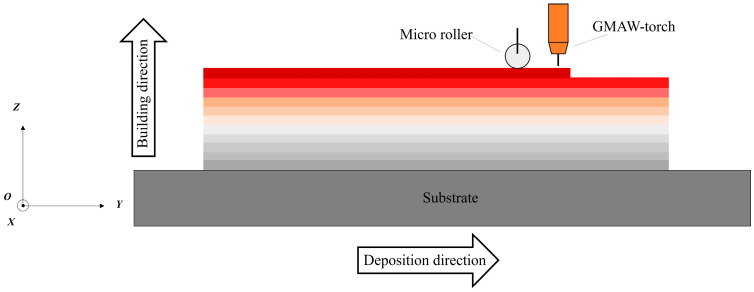
In situ rolling hybrid wire arc additive manufacturing AerMet100 steel process and defining direction.

The raw material used is 1.2 mm diameter AerMet100 steel wire. Before carrying out the experiments, ensure that the AerMet100 steel wire is intact and free from stains and cracks. The in situ rolling hybrid with wire arc additive manufacturing platform independently developed by Huazhong University of Science and Technology was used to complete the experiment. The welding machine is equipped with a Tetrix551 DC welding power. The self-developed control system ensures the multi-axis robot and micro-roller move synchronously with the welding gun. Figure 1 shows the schematic diagram and the definitions of deposition direction (DD), building direction (BD), and X, Y, and Z directions. The experiments were carried out in an enclosed chamber containing the welding torch, roller, and robotic arm, which were gas-protected with argon, in an oxygen content reduced to less than 0.004 vol.%. Based on previous experimental experience, a combination of process parameters was selected to provide good roll force, wire feed speed, travel speed, forming current, and inter-layer temperature. The process parameters are shown in Table 1. Thin-walled specimens with dimensions of 240 mm length × 15 mm width × 105 mm height were produced, the top surface of which is smooth and flat. A series of heat treatment processes and parameters are shown in Table 2. The material composition of the as-deposited AerMet100 steel is shown in Table 3, which conforms to the requirements of the standard.

### 2.2. Microstructural Characterization

Three planes perpendicular to the *Y*-axis, *X*-axis, and *Z*-axis of the as-deposited and heat-treated AerMet100 steel were chosen for microstructure examination. The cut surfaces were ground and, polished and then etched with a 4% nitric acid solution. The material microstructure was observed using a ZEISS SCOPE A1 optical microscope (OM), (Carl Zeiss, Oberkochen, Germany). The OXFORD Quanta 450 scanning electron microscope (SEM) (Oxford Instruments, Oxford, UK) was used to observe the fracture morphology. Electron backscattered diffraction (EBSD) was conducted using a GAIA3 GMU Model scanning electron microscope (TESCAN, Brno, Czech Republic) with a scanning step of 0.5 μm on heat-treated AerMet100 steel. Nanoscale microstructural features were investigated using a Talos F200X G2 transmission electron microscope (TEM) (Thermo Fisher Scientific, Waltham, MA, USA) on heat-treated AerMet100 steel.

### 2.3. Mechanical Property Tests and Fracture Analysis

Three tensile test rods were taken separately from as-deposited and heat-treated specimens in deposition direction and building direction. In accordance with GB/T 228.1-2010 [30], a bar specimen with a length of 75 mm and a diameter of 5 mm was used for the test. The test was performed using a CMT5305 universal electronic tensile testing machine at room temperature.

**Figure 2:**
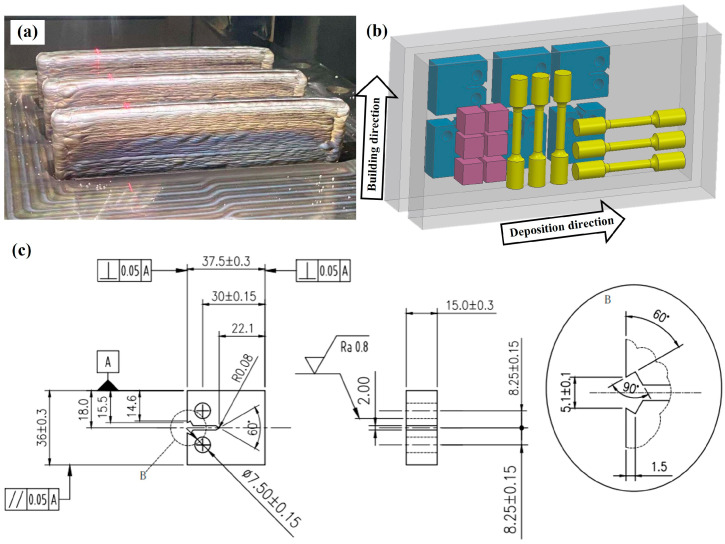
(**a**) Straight wall of AerMet100 steel produced using in situ rolling hybrid wire arc additive manufacturing; (**b**) sampling diagrams in different directions; (**c**) the size of the fracture toughness sample.

Plane strain fracture toughness testing was performed in accordance with the ASTM E399-20a standard [31]. After the heat treatment process, three compact tensile specimens were obtained for the plane strain fracture toughness test, separately in the deposition direction and the building direction. For each specimen, a fatigue pre-crack was made at approximately 3 mm from the pre-cracked notch prior to conducting the fracture toughness test. Crack opening displacement (COD) was then measured using a clip-on extensometer. Side grooves were also machined to ensure a flat crack front and to provide reliable crack length measurements. Plane strain fracture toughness testing was performed using the MTS Land Mark 50 KN hydraulic servo testing system. Standard procedures were followed to assess initial and final crack lengths. The average initial crack size was determined by measuring five equally spaced positions. Figure 2 displays the detailed dimensions of the tensile specimens and plane strain fracture toughness specimens. The fracture morphology was analyzed using an OXFORD Quanta 450 scanning electron microscopy.

## 3. Results

### 3.1. Microstructure Analysis

**Figure 3:**
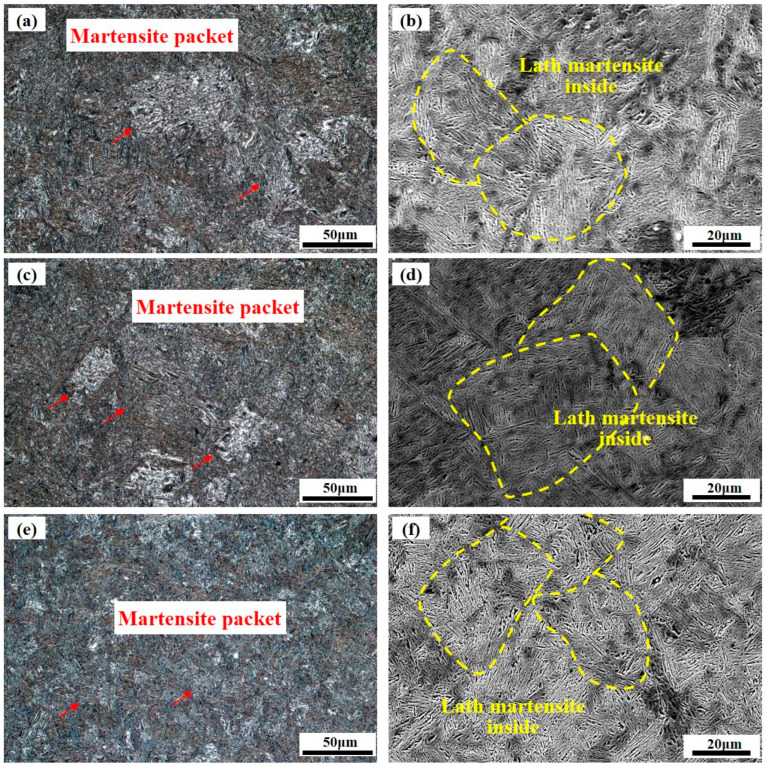
Microstructure of as-deposited AerMet100 steel fabricated using WAAM hybrid with in situ rolling: microstructure morphology in planes perpendicular to (**a**,**b**) *Y*-axis; (**c**,**d**) *X*-axis and (**e**,**f**) *Z*-axis.

Figure 3 shows the micromorphology in planes perpendicular to the *Y*-axis, *X*-axis, and *Z*-axis of the as-deposited AerMet100. The grain morphology appears to be highly irregular, and there is no obvious grain boundary in Figure 3a,c,e. Previous research has shown that AerMet100 steel produced using conventional additive manufacturing typically has a clear preferred orientation, and the material properties also show significant anisotropy [9]. There is no obvious difference in morphology and no obvious preferred orientation in the three directions. In this study, the in situ rolling hybrid with the WAAM process applies compressive stress to the molten pool that is not yet solidified, resulting in grain refinement and breakage, achieving the desired effect of grain refinement [21]. The microstructure of the AerMet100 steel is mainly composed of lath martensite with an irregular orientation. It can be seen in Figure 3b,d,f that a group of oriented lath martensite forms a martensite packet. There is the Kurdjumov–Sachs orientation relationship between prior austenite and martensite [14]. The most basic structural unit is lath martensite with a small angular deviation (2–5°). Lath martensite is mainly found in low-carbon steel, whereas the carbon content of AerMet100 steel is very low, only 0.21%. Figure 3b,d,f show preliminary judgments based on the micromorphology of as-deposited AerMet100 steel, with possible austenite grains circled, which probably have a grain size of around 40–60 μm.

**Figure 4:**
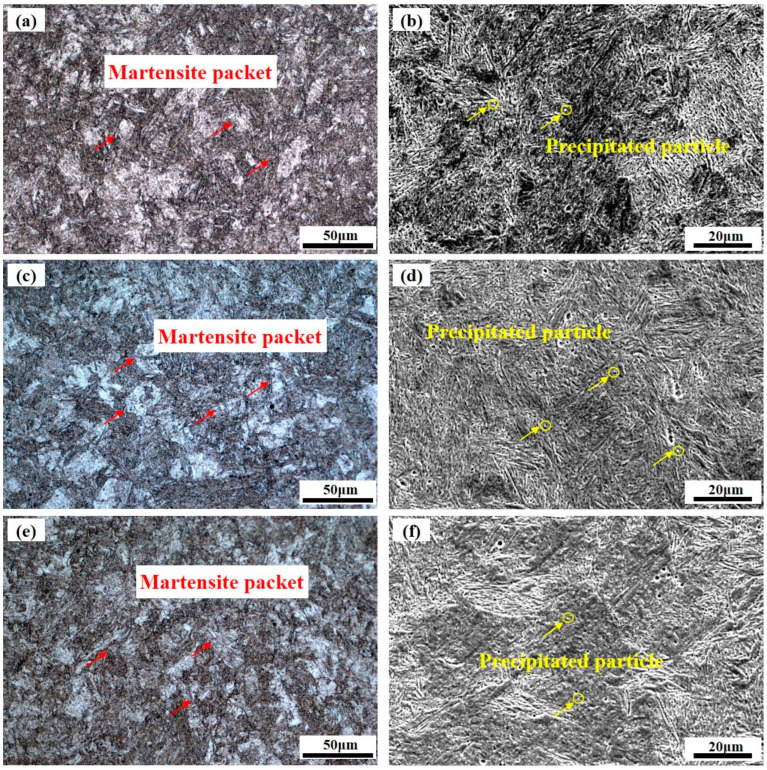
Microstructure morphology of AerMet100 after heat treatment produced using an in situ rolling hybrid WAAM process: microstructure morphology in planes perpendicular to the (**a**,**b**) *Y*-axis; (**c**,**d**) *X*-axis and (**e**,**f**) *Z*-axis.

The microstructure morphology of AerMet100 steel produced using an in situ rolling hybrid WAAM process after heat treatment is shown in Figure 4. As the original grains are crushed and refined using in situ rolling during the deposition process, the grain size of the AerMet100 steel remains small after heat treatment [32], and there is no difference in the microstructure in the three directions. In Figure 4a,c,e, it can be seen that the microstructure is still mainly composed of martensite packets, and according to previous research there may be a small amount of reversed austenite [33]. Figure 4b,d,f show that the fine particles are precipitated in the heat-treated AerMet100 steel during the heat treatment process [13], which can trap dislocations and increase the strength of the material [34].

### 3.2. Tensile Properties Analysis

**Figure 5:**
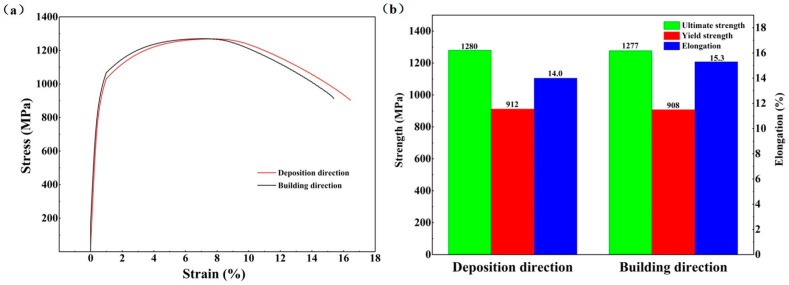
Tensile results of the as-deposited AerMet100 specimens in different directions: (**a**) stress–strain curves and (**b**) the corresponding ultimate tensile strength, yield strength, and elongation.

Figure 5 shows the tensile results of the as-deposited AerMet100 specimens in deposition and building directions. In Figure 5a, the tensile mechanical behavior of the as-deposited specimens in two directions showed the same trend. The curves in both directions are consistent and show no obvious difference. Figure 5b shows the ultimate tensile strength, yield strength, and elongation after fracture in the deposition direction, which are 1280 MPa, 912 MPa, and 14.0%, respectively. Meanwhile, these data in building direction are 1277 MPa, 908 MPa, and 15.3%. The tensile properties of the AerMet100 in building direction and deposition direction show little difference, which differs from traditional additive manufacturing [9]. The introduction of a microrolling process into each melting deposition layer can significantly prevent the development of columnar crystals [35], compact the pore defects, weaken the texture [10,36], promote the formation of equiaxed crystals, and increase the tensile strength of materials. This process can eliminate the differences in tensile properties between the deposition direction and the building direction. In conventional additive manufacturing, the grain aligns with the greatest temperature gradient, resulting in the development of columnar crystals following the direction of heat flow [37]. This phenomenon results in the tensile properties of additively manufactured materials showing significant variations in the deposition and building directions.

**Figure 6:**
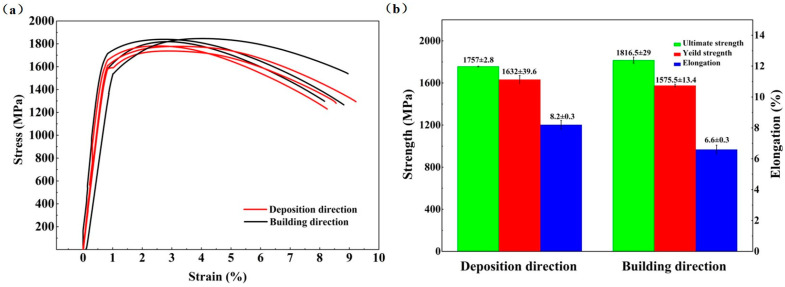
Tensile results of the heat-treated AerMet100 specimens in different directions: (**a**) stress–strain curves and (**b**) the corresponding ultimate tensile strength, yield strength, and elongation.

Figure 6 shows the tensile results of the heat-treated AerMet100 specimens in deposition and building directions. In Figure 6a, the tensile mechanical behavior of the heat-treated AerMet100 steel shows the same trend in both directions: the short uniform plastic deformation stage and the long local necking stage (after reaching the ultimate tensile strength) [34]. The stress–strain curves in the two directions are consistent and show no obvious difference. Figure 6b shows the ultimate tensile strength, yield strength, and elongation after fracture in deposition direction are 1747.7 ± 16.3 MPa, 1615 ± 40.6 MPa, and 8.3 ± 0.2%, respectively. Meanwhile, these data in building direction are 1821.3 ± 22.1 MPa, 1624 ± 84.5 MPa, 7.6 ± 1.7%. The tensile properties of the AerMet100 in building direction and deposition direction show little difference, and the ultimate tensile strength of the AerMet100 steel significantly improves after heat treatment compared with the as-deposited specimens. On the contrary, The AerMet100 steel, after heat treatment, displays a lower elongation compared with the as-deposited.

It is widely accepted that during the quenching process, lath martensite develops with high-density dislocations is formed. During the tempering process, many tiny and dispersed M_2_C carbides would be precipitated, which is the main mechanism for the secondary hardening effect of AerMet100 steel [13]. The high density of dislocations and dispersed M_2_C carbides in the matrix of the lath martensite leads to the high-level strength of this steel [38]. Furthermore, the crystal structure of the lath martensite enables several slip systems to be activated during deformation, which accounts for its plasticity behavior.

**Figure 7:**
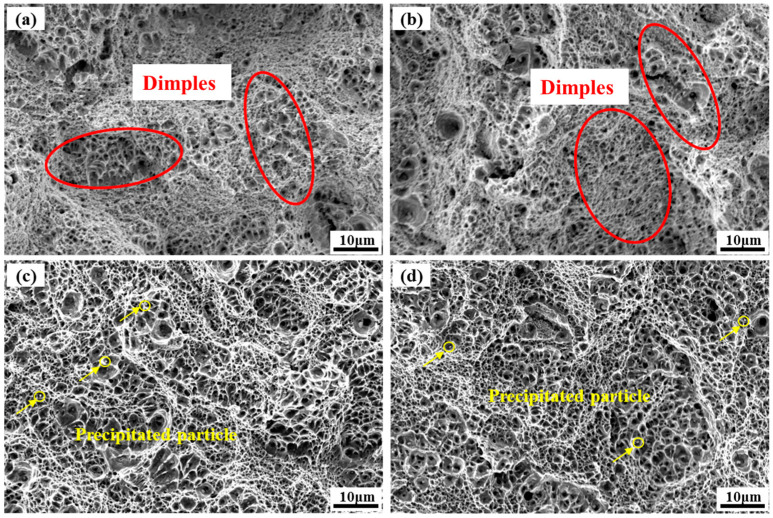
Tensile fracture morphology of AerMet100 steel in different directions: (**a**) as-deposited specimen in DD; (**b**) as-deposited specimen in BD; (**c**) heat-treated specimen in DD; (**d**) heat-treated specimen in BD.

The fracture morphology of the as-deposited and heat-treated AerMet100 tensile specimen in the deposition direction and building direction is shown in Figure 7. From the fracture morphology, it can be seen that both the as-deposited and heat-treated AerMet100 steel have a large number of dimples, indicating that the material is a ductile fracture. In addition, the fracture morphology of the as-deposited specimen is more uneven with tearing edges in Figure 7a,b, and the sizes of the dimples in the as-deposited specimen are smaller, indicating that the plasticity of the as-deposited specimen is better, which is consistent with the higher elongation compared with the heat-treated specimen. In Figure 7c,d, the second phase particles or carbides appear at the bottom of the dimples. In precipitation-hardened alloys, the presence of secondary nanoparticles could promote microvoid nucleation during tensile fracture. During the tensile loading process, the voids would aggregate and connect, ultimately leading to material fracture. As the aging process progresses, more secondary nanoparticles may be precipitated, resulting in the formation of more crack sources. Therefore, the elongation of AerMet100 steel in the heat-treated state decreases compared with that in the deposited state.

### 3.3. Plain Strain Fracture Toughness Test and Fracture

Results from the plane strain fracture toughness test of heat-treated AerMet100 are presented in Table 4, and all the tested specimens attained valid K_IC_ test results. The mean K_IC_ value for the deposition direction was 68.5 MPa/m^0.5^, while the value for the building direction was 59.25 MPa/m^0.5^. The K_IC_ values differ slightly in both directions, but not significantly.

**Figure 8:**
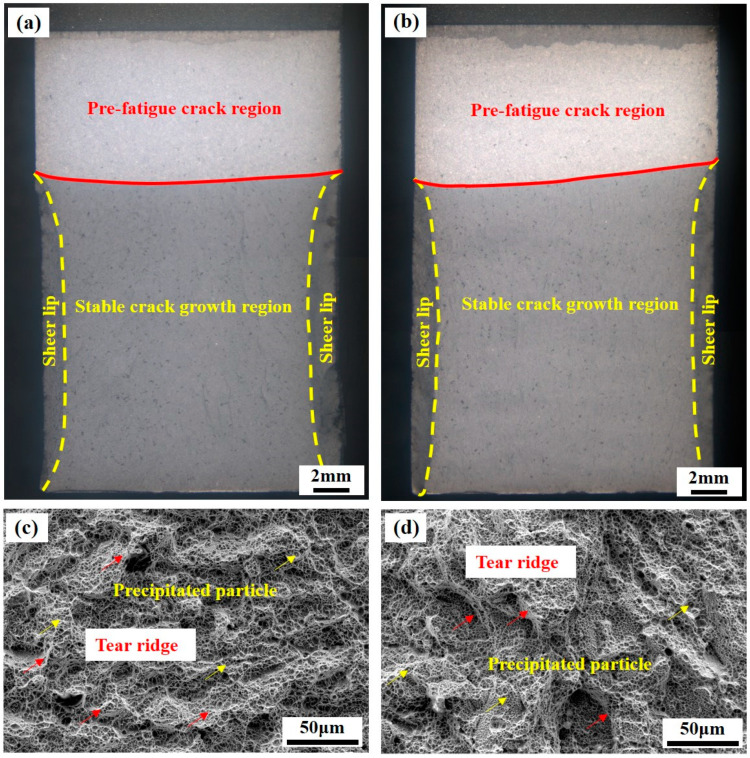
Fracture surface morphology of the plain–strain fracture toughness specimen: (**a**) macrostructure in DD; (**b**) macrostructure in BD; (**c**) microstructure in DD; (**d**) microstructure in BD.

Figure 8 shows the macroscopic and microscopic fracture morphology of the fracture toughness specimens in the deposition direction and building direction. As shown in Figure 8a,b, the macroscopic fracture surface morphology of the K_IC_ specimen consists of pre-fatigue fracture, stable crack growth regions, and sheer lip. The obviously shear lip can be observed, which illustrates the high toughness [39]. Scanning electron microscopy in Figure 8c,d can clearly distinguish the toughness dimples. The surfaces of the fracture are not smooth, with many tearing edges. Energy absorption during fracture is one of the measures used to evaluate fracture toughness. The microscopic fracture surface morphology of materials would, therefore, be used to judge fracture toughness [40].

## 4. Discussion

### 4.1. Effect of Hybrid Manufacturing Process on Strengthening of Mechanical Properties

**Figure 9:**
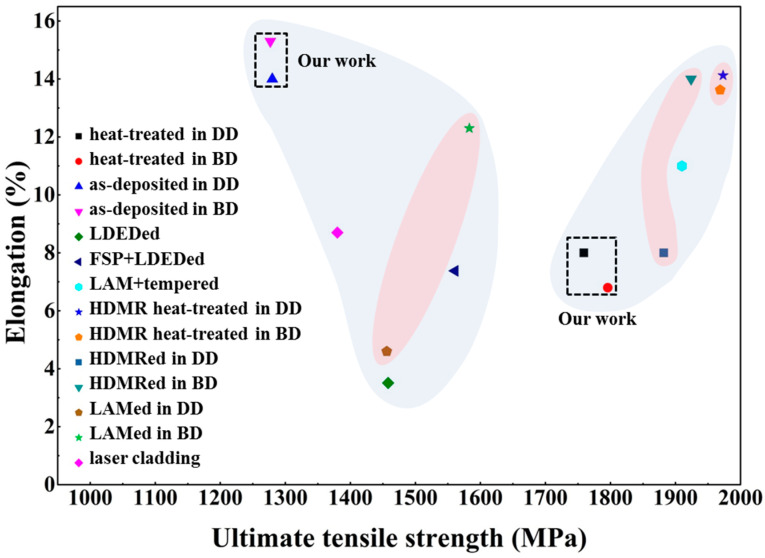
A summary chart of AerMet100 mechanical properties.

Figure 9 shows the summary chart of AerMet100 steel mechanical properties. The mechanical performance data associated with the additive manufacturing of AerMet100 steel are shown in Figure 9. Among them, the light blue background represents the performance of the deposited state and the heat-treated state. The light red background represents the mechanical properties in different directions under the same process conditions. It can be seen that the ultimate tensile strength of heat-treated AerMet100 steel is significantly improved compared with the as-deposited material. Our work has a high elongation in as-deposited, which is significantly higher than other manufacturing processes, which may be due to the grain refinement caused by the introduction of microrolling. Compared with the mechanical properties of the LAM process, which exhibit anisotropy in both the deposited and heat-treated states, it can be seen that our material is isotropic in the deposited and heat-treated states. Compared with Lu’s work [10], it can be seen that our work is isotropy in both deposited and heat-treated states, indicating that our process does not eliminate anisotropy due to heat treatment but rather introduces in situ microrolling manufacturing in the deposited state and maintaining isotropy after heat treatment.

In the tempering process, the microstructure evolution behavior of AerMet100 steel is dependent on austenite decomposition, martensite formation, M_2_C carbide formation and coarsening, and film-like reversed austenite formation and thickening [12]. The strengthening mechanism of AerMet100 steel can be divided into precipitation strengthening, grain boundary strengthening, and dislocation strengthening.

**Figure 10:**
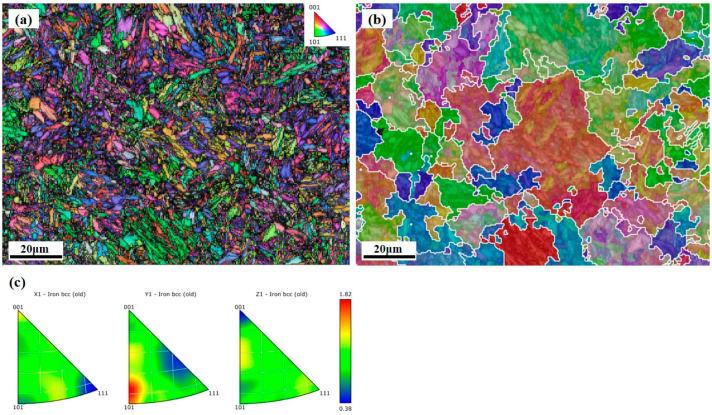
(**a**) Martensite lath in heat-treated AerMet100 steel; (**b**) austenite reconstruction of the microstructure in (**a**); (**c**) inverse pole figure of (**a**).

As can be seen in Figure 10a, a large number of lath martensites have different orientations within prior austenite grains. Due to grain fragmentation and random orientation, visualization of the austenite grains is challenging in the WAAM hybrid with an in situ rolling process [41]. AZtecCrystal software 2.1 was used to reconstruct the austenite grain based on the K-S orientation relationship for the martensite microstructure in the heat-treated AerMet100 steel. As can be seen in Figure 10b, the prior austenite grains are approximately 40–60 µm in size, and the grain shape is irregular. By means of the inverse pole figure map in Figure 10c, we can learn that although some grains have (101) directions parallel to the Y1 direction of the sample coordinate system, the selective orientation is not obvious due to Mmax = 1.82. There is no apparent texture visible in the sample shown in Figure 10. The process of utilizing in situ rolling hybrid with wire arc additive manufacturing proves to be effective in breaking up prior austenite grains and eliminating texture, leading to a random orientation distribution and no obvious anisotropy of the materials. According to the Hall–Petch equation [42,43]:

*σ_H_* = *σ*_0_ + k/*D*^1/2^ (1)

where *σ_H_* is the material strength, σ_0_ is the frictional stress, k is a constant, and *D* is the grain diameter. Based on this equation, *σ_H_* increases as the grain size decreases. Grain refinement can enhance mechanical properties attributable to the inhibition of dislocation movement by grain boundary interactions. Due to the different orientations of the grains on either side of the boundary, a stress field is generated when dislocations approach the boundary, hindering the movement of other dislocations. The smaller the grain size, the higher the stress required for dislocation movement in neighboring grains with different orientations. As the grain size decreases, the percentage of grain boundaries rises, potentially creating additional impediments for dislocation across these boundaries and enhancing the strength of the material [20].

**Figure 11:**
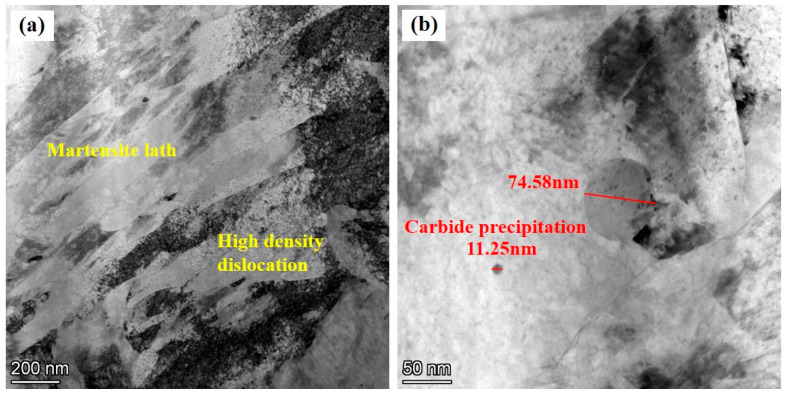
TEM photographs of heat-treated AerMet100 steel: (**a**) the lath martensite with high-density dislocation in it, (**b**) the carbide precipitation.

As shown in Figure 11a, the lath martensite structure of AerMet100 steel displays high-density dislocation. The strengthening process is also influenced significantly by carbide precipitation and dislocation [44]. The high-density dislocation entanglement in the AerMet100 steel hinders the movement of slip systems, making plastic deformation difficult and thus improving the strength [45,46]. Figure 11b shows that the precipitated particles are spheroidal distributed in the lath martensite. There are two kinds of precipitated particles: big spheroidal particles with a size of 74.58 nm and small-sized precipitated phases with a size of 11.25 nm. Nanoscale fine and dispersed carbide precipitates in lath martensite strengthen dislocation pinning and hinder the movement of the slip system, effectively forming dislocation delivery or twinning, thereby enhancing strength through precipitation strengthening [47,48,49,50]. The mechanism of precipitation enhancement depends on the size, density, and type of the nanoscale precipitate, in addition to the interaction between the dislocation and the precipitate [51]. For precipitation-hardened steel, there is an Orowan bypass hardening mechanism [52,53,54]. During the aging process, the martensite matrix is strengthened by the formation of uniform and dense alloy carbide particles, which act as precipitates. The Orowan strengthening mechanism indicates that dislocation lines cannot directly cut second-phase particles during plastic deformation, but external forces can bend around them. The bending of dislocation lines amplifies the lattice distortion energy in the dislocation region, leading to an increase in dislocation line resistance and slip resistance.

### 4.2. Fracture Behavior and Crack Propagation Mechanisms

As a result, similar fracture toughness values were observed in both the building direction and deposition direction. In addition, compressive residual stresses are beneficial in inhibiting crack initiation and early expansion, thus improving crack resistance [22].

Ductile fracture occured due to the growth and aggregation of micropore, resulting in slow and steady crack extension. This macroscopic fracture mode had a continuous process of ductile tearing, which absorbed more energy. Theoretically, the better the fracture toughness, the more tortuous the macroscopic fracture path of a specimen [39]. To better understand the crack extension mechanism, Figure 8 examines the macroscopic crack extension paths of fracture toughness specimens in both the deposition and building directions.

**Figure 12:**
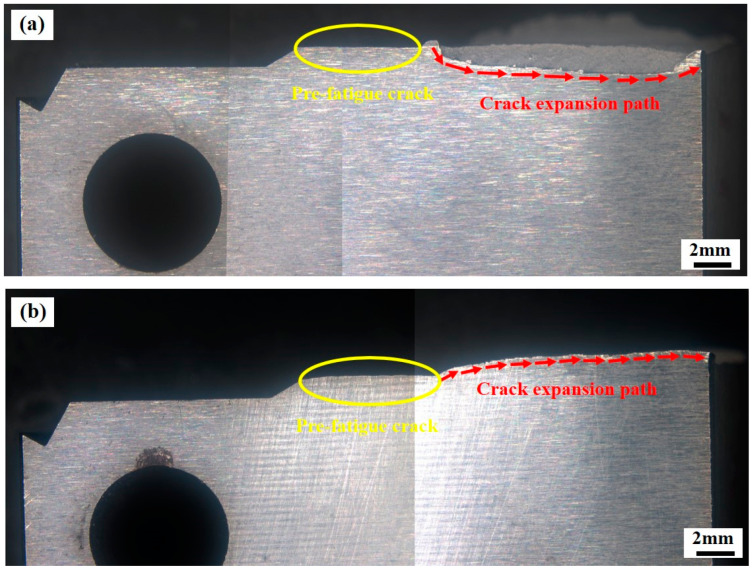
Crack expansion path: (**a**) in the deposition direction; (**b**) in the building direction.

The crack extension can be divided into two phases: the pre-fatigue crack and the crack instability extension. In the fatigue crack region, as shown in Figure 12a,b, the crack extension path is relatively flat. However, in the unstable stage, the angle of the crack extension path increases significantly, which means that the sample with high fracture toughness will experience greater plastic deformation during the fracture process.

**Figure 13:**
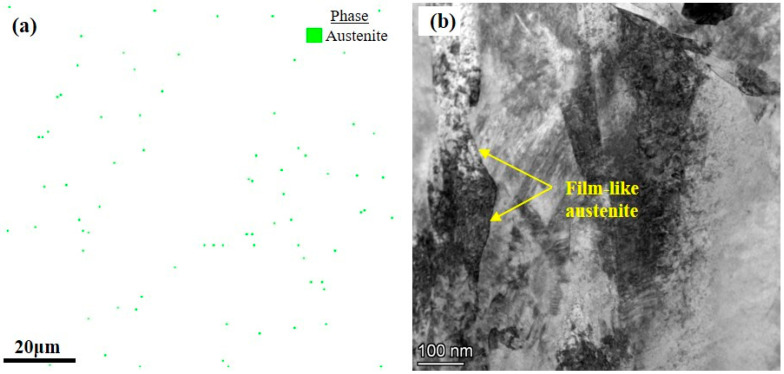
Film-like austenite distribution at the boundary of lath martensite in heat-treated AerMet100 steel: (**a**) EBSD phase composition characterization; (**b**) TEM morphology.

The morphology of the fracture surface is composed of small and large dimples, as shown in Figure 7. The dominant crack would join the microvoids near the crack tip in a stress-dominated mode, thereby propagating the crack rapidly. Meanwhile, the microvoids in front of the crack tip would grow and connect, causing the dominant to propagate in a strain-dominated manner, leading to ductile fracture [39]. Figure 13a demonstrates the phase composition of AerMet100 steel after heat treatment by EBSD analysis. A minimal proportion of reversed austenite is noticeable in the specimen, which correlates with the TEM discoveries in Figure 13b. Figure 13b presents a TEM bright field micrograph of heat-treated AerMet100 steel, showing film-like reversed austenite in the martensite interlayers of the steels. During the tempering at 482 °C, the continuous film of reversed austenite started to precipitate at lath martensite boundaries. It has been reported that fine film-like reversed austenite prevents crack initiation and propagation by deformation and transformation [55], slowing the rate of crack extension in steel, releasing stress conditions, and blunting the crack tip, resulting in higher resistance to crack extension [56]. On the one hand, the existence of an austenite film between the lath martensite can decrease the possibility of local stress concentration and the potential for micro-crack formation during the deformation of AerMet100 steel, thus significantly improving the plasticity [2]. On the other hand, due to the excellent flexibility and stability of the reversed austenite, fracture toughness is improved by promoting the “zigzag” path of crack deflection [15].

## 5. Conclusions

In this study, AerMet100 steel was produced using an in situ rolling hybrid with a wire arc additive manufacturing process. The microstructure, tensile properties, and fracture toughness of the as-deposited and heat-treated AerMet100 steel were evaluated in different directions. The results show that our method can not only produce AerMet100 steel with isotropic mechanical properties but also achieve near-net forming and reduced machining, which can be used for the production of parts with complex shapes and relatively high mechanical property requirements, such as landing gears.

(1) The primary microstructure of the AerMet100 steel produced using in situ rolling hybrid with wire arc additive manufacturing is a martensite packet with lath martensite inside, and after heat treatment, the martensite, carbides precipitation, and the film-like austenite. The microstructure and mechanical properties of the AerMet100 steel show isotropy in as-deposited state and remain isotropy after heat treatment.
(2) The AerMet100 steel exhibited ductile fracture, with micro-hole nucleation and coalescence being the predominant mechanisms. In addition, alloy carbide was precipitated in the matrix, and the strength of the material was enhanced by the precipitation-strengthening mechanism. This material exhibits isotropic tensile properties, where the ultimate tensile strength, yield strength, and elongation after fracture in the deposition direction are 1747.7 ± 16.3 MPa, 1615 ± 40.6 MPa, and 8.3 ± 0.2%, respectively, while the corresponding values in the building direction are 1821.3 ± 22.1 MPa, 1624 ± 84.5 MPa, and 7.6 ± 1.7%.
(3) The austenite film, the film-like structure formed by tempering at 482 °C, is located among the martensite matrix, which can turn the crack growth path and absorb the energy consumed in the crack growth process, thus playing a role in increasing the toughness and improving the fracture toughness to 70.6 MPa/m^0.5^.

## Figures and Tables

**Table 1:** Additive manufacturing parameters for in situ rolling hybrid with WAAM.

Parameter	Value
Preheating temperature (°C)	100
Roll force (KN)	10
Wire feed speed (m/min)	1.3
Travel speed (mm/min)	120
Voltage (V)	23
Current (A)	200
Gas flow (L/min)	15

**Table 2:** Process parameters for heat treatment.

Process	Parameter
Normalizing	900 °C × 1 h, Air cooling
High-temperature tempering	680 °C × 16 h, Air cooling
Quenching	885 °C × 1 h, Oil cooling
Cryogenic treatment	−73 °C × 1 h, Warming in the air
Tempering	482 °C × 5 h, Air cooling

**Table 3:** Chemical composition of as-deposited AerMet100 steel samples.

Elements	C	S	P	Si	Mo	Cr	Ni
Content (wt.%)	0.21	0.019	0.010	0.04	1.16	2.94	10.57
Elements	Ti	Al	Mn	Co	O	N	Fe
Content (wt.%)	0.01	0.01	0.03	14.13	0.001	0.0004	Balance

**Table 4:** Plain–strain fracture toughness properties of AerMet100 steel after heat treatment fabricated using an in situ rolling hybrid WAAM process.

Sample Direction	K_IC_ (MPa/m^0.5^)
Deposition direction	70.6
	66.4
Building direction	58.5
	60

## Data Availability

Data are contained within the article.
